# Loss of LLGL1 Elevates EGFR/RAS/MAPK Signaling and Remodels EMT Markers in Huh-7 Hepatocellular Carcinoma Cells

**DOI:** 10.3390/ijms27072959

**Published:** 2026-03-24

**Authors:** Gökhan Yıldız, Soner Karabulut, Tuba Dinçer, Bayram Toraman

**Affiliations:** 1 Department of Medical Biology, Faculty of Medicine, Karadeniz Technical University, 61080 Trabzon, Türkiye; tdincer@ktu.edu.tr (T.D.); bayramtoraman@yahoo.com (B.T.); 2 Department of Medical Biology, Graduate School of Health Sciences, Karadeniz Technical University, 61080 Trabzon, Türkiye; soner_karabulut45@hotmail.com

**Keywords:** cell invasion, cell migration, EGFR/RAS/MAPK signaling, epithelial cell polarity, epithelial–mesenchymal plasticity, hepatocellular carcinoma, LLGL1

## Abstract

Loss of epithelial polarity is a critical driver of tumor progression; however, how core polarity regulators interface with oncogenic signaling pathways in hepatocellular carcinoma (HCC) remains incompletely defined. LLGL scribble cell polarity complex component 1 (LLGL1) is an evolutionarily conserved polarity protein with well-established tumor-suppressive roles in multiple epithelial malignancies. Nevertheless, how LLGL1 loss shapes oncogenic signaling outputs and cellular phenotypes in HCC remains unclear. In this study, we investigated the consequences of LLGL1 knockout (KO) in epithelial-like Huh-7 HCC cells. LLGL1 loss resulted in enhanced proliferative capacity and increased clonogenic potential, accompanied by altered cell-cycle distribution characterized by reduced G1-phase and increased S-phase fractions (*p* < 0.001). At the signaling level, LLGL1 KO cells displayed potentiated EGFR-driven RAS/MAPK pathway activation, with increased EGFR phosphorylation, enhanced downstream RAF1–MEK–ERK–RSK signaling, elevated EGFR abundance, and selective modulation of RAF1 protein levels. Functionally, LLGL1 loss markedly enhanced migratory and invasive behavior (*p* < 0.0001). Despite increased motility, LLGL1 KO cells exhibited remodeling of epithelial–mesenchymal transition (EMT)-associated markers without evidence of a classical EMT program. Collectively, these findings position LLGL1 loss as a central factor associated with altered MAPK signaling, EMT marker remodeling, and tumor-promoting cellular phenotypes in HCC.

## 1. Introduction

Epithelial cell polarity is a fundamental determinant of tissue architecture, ensuring spatial organization of membrane domains, preservation of cell–cell junctions, and coordinated cell behavior [1]. Beyond maintaining epithelial architecture, polarity programs actively shape intracellular signaling pathways that regulate proliferation, differentiation, and survival [2]. Disruption of epithelial polarity leads to deregulated signaling and loosening of epithelial growth constraints, thereby creating a permissive environment for oncogenic transformation [2]. Accordingly, loss of epithelial polarity is increasingly recognized as a key driver of cancer progression and malignant cell behavior [3].

LLGL scribble cell polarity complex component 1 (*LLGL1*; Gene ID: 3996) is an evolutionarily conserved regulator of epithelial organization and polarity. It is the mammalian ortholog of *Drosophila* lethal(2) giant larvae, a protein originally characterized as a tumor suppressor essential for epithelial integrity and growth control [4]. In mammalian epithelial cells, LLGL1 predominantly localizes to basolateral membrane domains, where it contributes to junctional stability, cytoskeletal coordination, and apicobasal polarity [5,6]. In line with these functions, reduced LLGL1 expression is associated with loss of epithelial polarity, increased proliferation, enhanced migratory and invasive behavior, and poor clinical outcome across diverse epithelial cancer types [7,8,9,10]. Conversely, restoration of LLGL1 suppresses tumor progression by reinforcing cell–cell adhesion and limiting invasive behavior [7,8,11]. Despite strong evidence supporting a tumor-suppressive role for LLGL1, the signaling mechanisms by which its loss is translated into oncogenic phenotypes remain poorly understood.

Building on these observations, the molecular mechanisms linking LLGL1 loss to oncogenic phenotypes have not been fully elucidated. The epidermal growth factor receptor (EGFR) is a major upstream activator of the RAS/mitogen-activated protein kinase (MAPK) signaling cascade, classically transmitted through the RAS–RAF–MEK–ERK axis, to regulate cell proliferation, survival, and motility [12]. Loss of LLGL1 has been reported to perturb EGFR localization and to coincide with increased ERK1/2 activation in an EGF-dependent setting in mammary epithelial cells [13]. In pancreatic ductal adenocarcinoma, LLGL1 has likewise been linked to altered ERK2 activity in the context of therapeutic response [14]. Collectively, these findings implicate LLGL1 as a regulator of EGFR–ERK signaling output. However, the impact of LLGL1 loss on EGFR/RAS/MAPK signaling has not yet been systematically characterized. In hepatocellular carcinoma (HCC), aberrant splicing of LLGL1 has been associated with disease progression, suggesting that LLGL1 dysregulation may contribute to liver cancer biology [15].

HCC accounts for approximately 80–90% of primary liver malignancies and ranks as the sixth most commonly diagnosed cancer and the third leading cause of cancer-related mortality worldwide [16]. Hepatocellular carcinogenesis is a multistep process characterized by progressive loss of epithelial differentiation and the gradual acquisition of aggressive cellular phenotypes [17]. Although advanced HCC often exhibits mesenchymal-like features, invasive and metastatic behavior can emerge through intermediate epithelial–mesenchymal states rather than through a uniform classical epithelial–mesenchymal transition (EMT) program [17]. In parallel, aberrant activation of the RAS/MAPK signaling pathway, a central regulator of cell proliferation, survival, migration, and epithelial plasticity, is frequently observed in HCC and contributes to tumor progression and therapeutic resistance [17,18,19]. Despite the pivotal roles of epithelial–mesenchymal plasticity (EMP) and RAS/MAPK signaling in HCC pathobiology, how epithelial organization and polarity regulators influence these processes remains poorly understood.

In this context, LLGL1 emerges as a compelling molecular link among epithelial organization, oncogenic signaling, and malignant cell behavior in HCC. We hypothesized that loss of LLGL1 amplifies EGFR/RAS/MAPK signaling and promotes invasive phenotypes by altering epithelial plasticity. To test this hypothesis, we systematically examined the functional consequences of LLGL1 loss in Huh-7 cells, focusing on growth control, EGFR/RAS/MAPK signaling activity, and migratory behavior. Through this study, we aimed to define how loss of a core epithelial organization and polarity regulator influences cellular signaling and phenotypic behavior in Huh-7 HCC cells.

## 2. Results

### 2.1. Generation of LLGL1 Knockout (KO) Huh-7 Cells and Analysis of Proliferative Phenotypes

**Figure 1:**
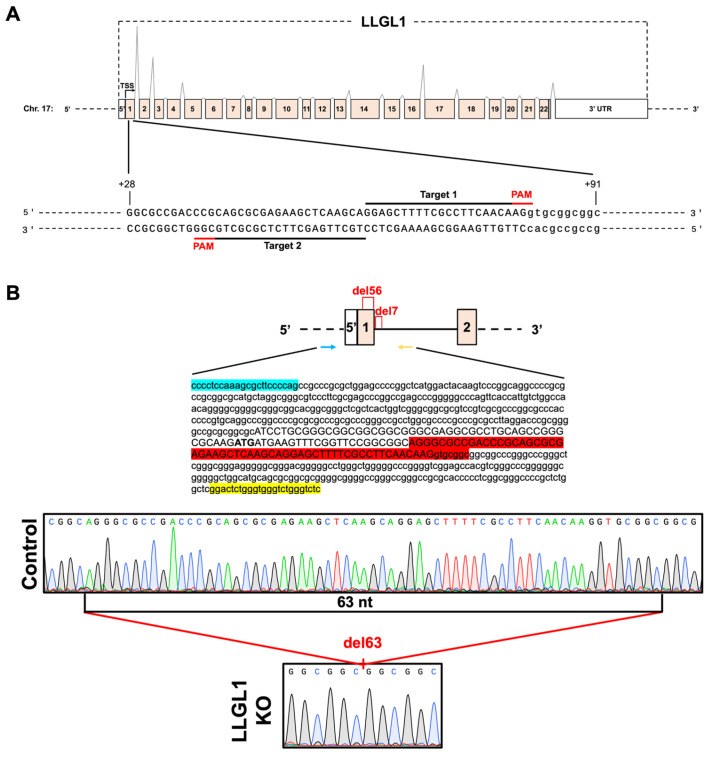
Generation and genetic validation of CRISPR/Cas9n-edited Huh-7 cells. (**A**) Schematic representation of the human *LLGL1* gene structure highlighting exon–intron organization. Exon 1, which was targeted for CRISPR/Cas9n-mediated genome editing, is indicated. The positions of the two guide RNAs (gRNAs) flanking the target region and the corresponding protospacer adjacent motif (PAM) sequences are shown. (**B**) Sanger sequencing analysis of the CRISPR/Cas9n target region in control and LLGL1 KO Huh-7 cells. Sequence alignment of control cells shows an intact *LLGL1* exon 1 sequence with a homozygous wild-type (WT) genotype. In contrast, edited Huh-7 cells harbor a homozygous deletion at the *LLGL1* target locus. Representative Sanger sequencing chromatograms of control and CRISPR/Cas9n-edited cells are shown at the bottom. Primer binding sites are indicated by colored arrows (blue, forward; yellow, reverse). The deleted sequence is highlighted in red on the partial sequence of the *LLGL1* gene. TSS: transcription start site; nt: nucleotide.

LLGL1 KO Huh-7 cells were generated by targeting exon 1 of the human *LLGL1* gene using a CRISPR/Cas9 nickase (CRISPR/Cas9n)-based genome-editing approach. Two guide RNAs (gRNAs) flanking the selected target region were designed, and their positions with the corresponding protospacer adjacent motif (PAM) sequences are schematically shown in Figure 1A. Sanger sequencing analysis of PCR products from control Huh-7 cells confirmed a homozygous wild-type (WT) sequence. In contrast, LLGL1 KO Huh-7 cells contained a homozygous 63-nucleotide genomic deletion (NC_000017.11:g.18225708_18225770del) at the CRISPR/Cas9n target locus (Figure 1B). This deletion comprised 56 nucleotides within exon 1 and an additional seven nucleotides extending into intron 1, resulting in disruption of the first exon and the exon–intron boundary (NM_004140.4:c.26_81+7del). These results confirm effective CRISPR/Cas9n-mediated disruption of the *LLGL1* genomic locus in Huh-7 cells.

**Figure 2:**
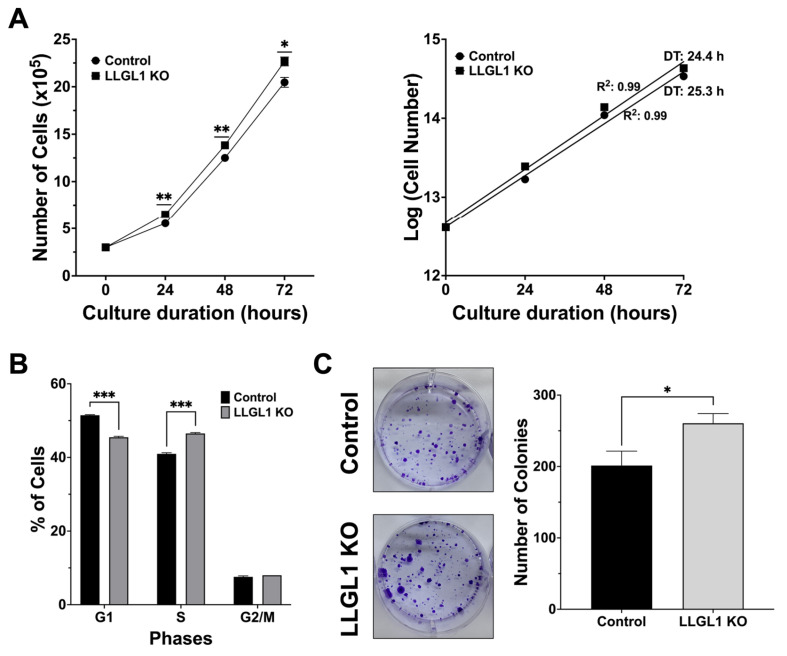
Loss of LLGL1 enhances proliferative and clonogenic properties of Huh-7 cells. (**A**) Population growth analysis of control and LLGL1 KO Huh-7 cells. The left panel shows time-course changes in cell numbers over 72 h, whereas the right panel shows population doubling times (DT) calculated by fitting growth curves to an exponential growth model using log-transformed population values. Statistical analyses were performed on cell number data shown in the left panel using multiple unpaired *t*-tests with Holm–Šidák correction for multiple comparisons. DTs shown in the right panel were calculated from the corresponding growth curves. (**B**) Cell-cycle distribution of control and LLGL1 KO Huh-7 cells determined by PI staining followed by flow cytometric analysis, showing the percentage of cells in G1, S, and G2/M phases. Phase-specific differences were evaluated using Welch’s unpaired *t*-test. (**C**) Representative images (**left**) and quantification (**right**) of colony formation assays performed in control and LLGL1 KO Huh-7 cells. Colony numbers were analyzed using an unpaired two-tailed *t*-test. Data are presented as mean ± SEM. *: *p* < 0.05, **: *p* < 0.01, ***: *p* < 0.001.

Having established LLGL1 KO Huh-7 cells, the impact of LLGL1 loss on cellular proliferation was assessed under standard culture conditions. Time-course growth analysis over 72 h showed consistently higher cell numbers in LLGL1 KO cultures compared with control cells (Figure 2A, left panel). Despite identical initial seeding densities, LLGL1 KO cells reached significantly higher cell numbers at 24 h (6.50 ± 0.06 × 10^5^ vs. 5.53 ± 0.12 × 10^5^ cells, 1.18-fold, *p* = 0.0089), 48 h (13.83 ± 0.12 × 10^5^ vs. 12.50 ± 0.21 × 10^5^ cells, 1.11-fold, *p* = 0.0089), and 72 h (22.67 ± 0.47 × 10^5^ vs. 20.47 ± 0.52 × 10^5^ cells, 1.11-fold, *p* = 0.0347).

Population doubling times (DT) were calculated by fitting the growth curves to an exponential growth model using log-transformed population values (Figure 2A, right panel). LLGL1 KO cells exhibited a shorter DT compared with control cells (24.4 h vs. 25.3 h, 0.96×, corresponding to a 3.6% decrease), consistent with the increased accumulation of cell numbers over time. Collectively, these findings demonstrate that LLGL1 loss promotes population expansion in Huh-7 cells.

To determine whether enhanced proliferation was associated with altered cell-cycle dynamics, cell-cycle distribution was analyzed by propidium iodide (PI) staining followed by flow cytometry (Figure 2B). LLGL1 KO cells displayed a significant reduction in the G1-phase population compared with control cells (45.49 ± 0.23% vs. 51.47 ± 0.15%, −5.98 percentage points, *p* = 0.0001), accompanied by a corresponding increase in the S-phase fraction (46.51 ± 0.23% vs. 40.96 ± 0.31%, +5.55 percentage points, *p* < 0.0001). In contrast, the proportion of cells in the G2/M phase did not differ significantly between LLGL1 KO and control cells (8.00 ± 0.00% vs. 7.57 ± 0.25%, +0.43 percentage points, *p* = 0.6019) (Figure 2B). These data indicate that LLGL1 loss is associated with an increased S-phase fraction without detectable changes in the G2/M-phase distribution.

To further evaluate the functional consequences of enhanced proliferative capacity, clonogenic growth was assessed by colony formation assays (Figure 2C). LLGL1 KO cells formed a significantly greater number of colonies compared with control cells (260.6 ± 13.5 vs. 201.3 ± 20.1 colonies, 1.29-fold, *p* = 0.0262), consistent with an increased clonogenic capacity under anchorage-dependent conditions.

### 2.2. LLGL1 Loss Potentiates EGFR/RAS/MAPK Signaling in Huh-7 Cells

**Figure 3:**
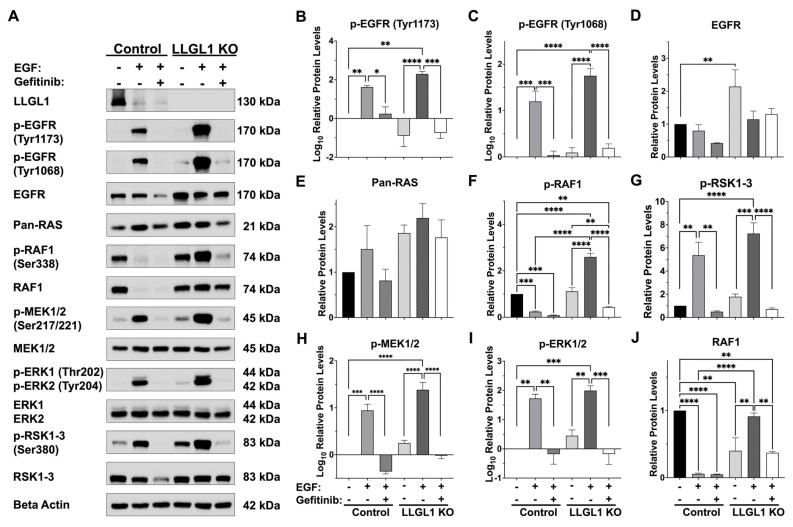
EGFR/RAS/MAPK signaling profiles in control and LLGL1 KO Huh-7 cells. (**A**) Representative Western blot images showing LLGL1 expression and key components of the EGFR–RAS/MAPK pathway proteins in control and LLGL1 KO Huh-7 cells under basal conditions and following EGF and/or gefitinib treatment. (**B**–**J**) Densitometric quantification of Western blot bands, normalized to the corresponding Beta Actin levels and expressed relative to untreated control cells. Phosphorylations of EGFR at Tyr1173 (**B**) EGFR at Tyr1068 (**C**), MEK1/2 at Ser217/Ser221 (**H**), and ERK1/2 at Thr202/Tyr204 (**I**) were analyzed and presented as log_10_-transformed fold changes relative to control cells to ensure data normality and variance homogeneity. In contrast, total protein levels of EGFR (**D**), pan-RAS (**E**) and RAF1 (**G**), as well as RAF1 phosphorylation at Ser338 (**F**) and RSK1–3 phosphorylation at Ser380 (**J**), are shown as relative protein levels normalized to control cells. Data are presented as mean ± SEM from three independent experiments. Statistical significance was determined by one-way analysis of variance (ANOVA) followed by Tukey’s multiple-comparisons post hoc test. *: *p* < 0.05, **: *p* < 0.01, ***: *p* < 0.001, ****: *p* < 0.0001.

EGFR/RAS/MAPK signaling was analyzed in control and LLGL1 KO Huh-7 cells by Western blotting to evaluate pathway modulation following LLGL1 loss (Figure 3). LLGL1 protein was undetectable by Western blotting in LLGL1 KO cells under all experimental conditions, indicating effective loss of LLGL1 protein expression (Figure 3A). Upon EGF stimulation, LLGL1 KO cells exhibited enhanced EGFR activation compared with control cells. Phosphorylation of EGFR at Tyr1173 increased to a log_10_ fold change of 2.30 in LLGL1 KO cells (*p* < 0.01, Figure 3B), compared with a log_10_ fold change of 1.62 in control cells (*p* < 0.05, Figure 3B). EGFR phosphorylation at Tyr1068 increased to a log_10_ fold change of 1.75 in LLGL1 KO cells (*p* < 0.0001, Figure 3C), whereas control cells showed a log_10_ fold change of 1.20 (*p* < 0.001, Figure 3C). Both phosphorylation events were attenuated by gefitinib treatment (Figure 3B,C). In addition to enhanced phosphorylation, total EGFR abundance increased by 2.14-fold relative to control in LLGL1 KO cells (*p* < 0.01, Figure 3D), whereas total pan-RAS levels did not differ significantly between groups (*p* > 0.05, Figure 3E). To assess whether increased total EGFR abundance was accompanied by elevated receptor presence at the plasma membrane, EGFR localization was further examined by confocal immunofluorescence (IF) under non-permeabilized conditions. Under identical imaging settings, LLGL1 KO cells displayed increased membrane-associated EGFR signal compared with control cells (Figure S1).

Downstream analyses further revealed altered regulation of RAF1 upon LLGL1 loss. Following EGF stimulation, RAF1 phosphorylation at Ser338 increased to 2.60-fold in LLGL1 KO cells (*p* < 0.0001, Figure 3F), whereas it was reduced to 0.24-fold in control cells (Figure 3F). Total RAF1 protein levels were strongly reduced in control cells (0.056-fold, *p* < 0.0001), while RAF1 abundance was partially maintained in LLGL1 KO cells under the same condition (0.913-fold, *p* < 0.01). A similar pattern was observed upon combined EGF and gefitinib treatment (Figure 3G). Consistent with increased upstream signaling, MEK1/2 phosphorylation at Ser217/Ser221 increased to a log_10_ fold change of 1.39 in LLGL1 KO cells (*p* < 0.0001, Figure 3H), whereas EGF stimulation resulted in a log_10_ fold change of 0.95 in control cells (*p* < 0.001, Figure 3H). ERK1/2 phosphorylation at Thr202/Tyr204 increased in both groups, reaching log_10_ fold change of 1.99 in LLGL1 KO cells and 1.73 in control cells (both *p* < 0.01, Figure 3I). Total MEK1/2 and ERK1/2 protein levels were not detectably altered (Figure 3A). RSK1–3 phosphorylation at Ser380 increased to 7.24-fold in LLGL1 KO cells (*p* < 0.001), compared with 5.37-fold in control cells (*p* < 0.01) and was attenuated by gefitinib (Figure 3J). Collectively, these data demonstrate enhanced EGFR-driven MAPK pathway signaling in LLGL1 KO Huh-7 cells without broad changes in total pathway component abundance.

### 2.3. LLGL1 Ablation Enhances Migratory and Invasive Capacities of Huh-7 Cells

**Figure 4:**
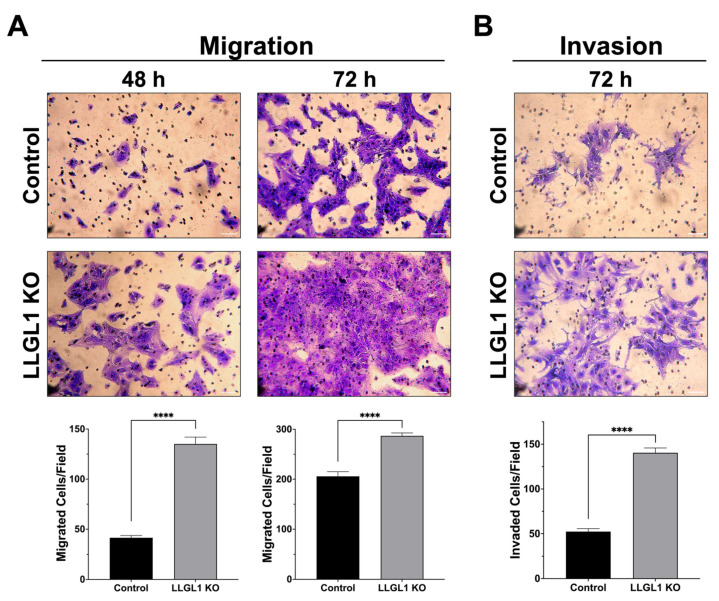
LLGL1 loss enhances migration and invasion of Huh-7 cells. (**A**) Representative images of migrated Huh-7 cells following transwell migration assays performed for 48 h and 72 h in control and LLGL1 KO cells. (**B**) Representative images of invading Huh-7 cells obtained from Matrigel-coated transwell invasion assays conducted for 72 h in control and LLGL1 KO cells. Images were acquired using a 10× objective with a scale bar of 200 µm. Membrane pores appear as unstained circles. Quantitative analyses were based on three independent biological experiments performed on different days. For each experiment, migrated or invaded cells were quantified from five randomly selected microscopic fields per transwell insert, and field-level counts were averaged to obtain a single value per experiment. Cell numbers were quantified using ImageJ software. Data are presented as mean ± SEM. Statistical significance was assessed using an unpaired two-tailed *t*-test with Welch’s correction. ****: *p* < 0.0001.

Transwell migration and invasion assays were performed to compare the migratory and invasive behavior of control and LLGL1 KO Huh-7 cells (Figure 4). Quantitative analysis revealed a significant increase in the migratory capacity of LLGL1 KO cells compared with control cells at both examined time points (Figure 4A). At 48 h, LLGL1 KO cells exhibited a 3.2-fold increase in migration, with a mean of 135.1 migrated cells per field compared with 41.7 cells per field in control cells (*p* < 0.0001). At 72 h, migration remained significantly elevated in LLGL1 KO cells, with 286.8 migrated cells per field compared with 205.8 cells per field in controls, corresponding to a 1.4-fold increase (*p* < 0.0001, Figure 4A). In Matrigel-based transwell invasion assays conducted over 72 h, LLGL1 KO cells showed a significant increase in invasive capacity compared with control cells (Figure 4B). LLGL1 KO cells displayed a 2.7-fold increase in invasion, with 140.5 invading cells per field versus 52.3 cells per field in control cells (*p* < 0.0001, Figure 4B). Collectively, these results indicate enhanced migratory and invasive capacities in LLGL1 KO Huh-7 cells.

### 2.4. LLGL1 Loss Remodels EMT-Associated Marker Proteins in Huh-7 Cells

**Figure 5:**
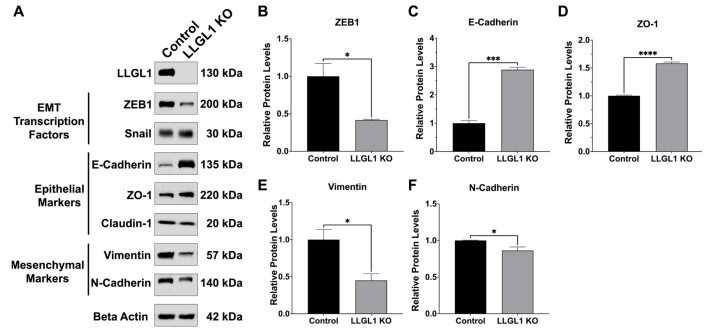
EMT-associated protein levels in control and LLGL1 KO Huh-7 cells. (**A**) Representative Western blot images showing LLGL1 protein levels, EMT-associated transcription factors (ZEB1 and Snail), epithelial junctional proteins (E-Cadherin, ZO-1, and Claudin-1), and mesenchymal markers (Vimentin and N-Cadherin) in control and LLGL1 KO Huh-7 cells. Beta Actin was used as the loading control. Representative blots are shown, and densitometric quantification includes all replicates. (**B**–**F**) Densitometric quantification of Western blot band intensities normalized to the corresponding Beta Actin signals and expressed relative to control cells for (**B**) ZEB1, (**C**) E-Cadherin, (**D**) ZO-1, (**E**) Vimentin, and (**F**) N-Cadherin. Data are presented as mean ± SEM from three independent biological replicates (n = 3). Statistical significance was determined using unpaired two-tailed *t*-tests. * *p* < 0.05, *** *p* < 0.001, **** *p* < 0.0001.

EMT-associated protein levels were examined in control and LLGL1 KO Huh-7 cells by Western blot analysis to evaluate molecular changes associated with LLGL1 loss (Figure 5). The marker set assessed here focused on core junctional and mesenchymal proteins and selected EMT-associated transcriptional regulators. Among the EMT-associated transcriptional regulators examined, ZEB1 abundance was reduced to 0.42-fold of control levels in LLGL1 KO cells (*p* = 0.0262, Figure 5B). Analysis of epithelial junctional proteins revealed increased protein levels of E-Cadherin and ZO-1 in LLGL1 KO cells. E-Cadherin levels increased to 2.89-fold of control (*p* = 0.0001, Figure 5C), while ZO-1 abundance increased to 1.58-fold of control (*p* < 0.0001, Figure 5D). Claudin-1 expression was reduced to 0.69-fold of control levels; however, the difference did not reach statistical significance (*p* = 0.0608, Figure 5A). Mesenchymal marker protein levels also differed between groups. Vimentin protein levels were reduced to 0.45-fold of control (*p* = 0.0293, Figure 5E), while N-Cadherin levels decreased to 0.86-fold of control (*p* = 0.0485, Figure 5F).

**Figure 6:**
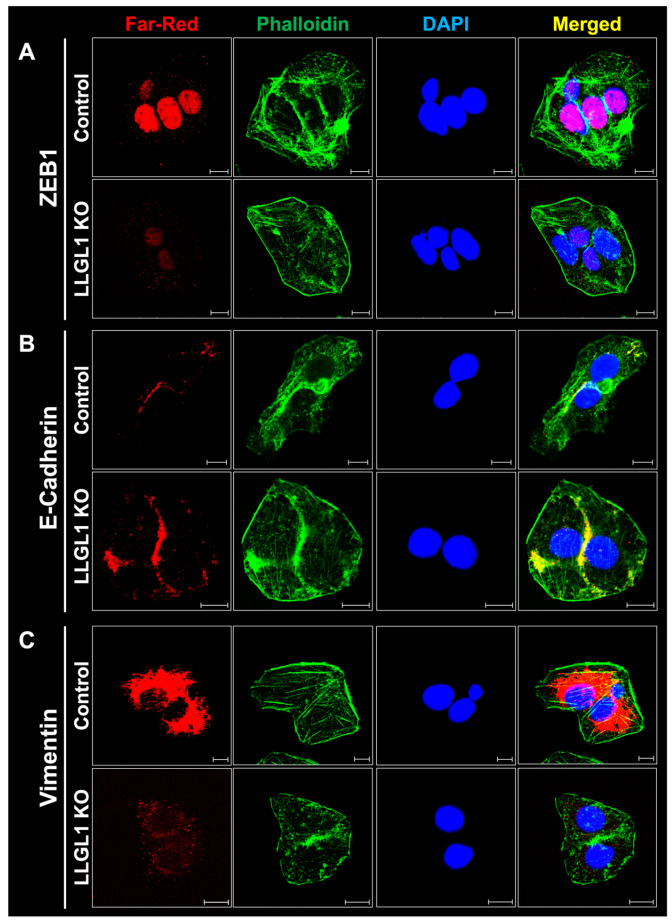
IF analysis of EMT-associated proteins in control and LLGL1 KO Huh-7 cells. (**A**–**C**) Representative confocal IF images showing the subcellular distribution of (**A**) ZEB1, (**B**) E-Cadherin, and (**C**) Vimentin in control and LLGL1 KO Huh-7 cells. Target proteins were detected using specific primary antibodies and far-red-emitting secondary antibodies (red). F-actin was visualized by co-staining with Alexa Fluor 488-conjugated phalloidin (green), and nuclei were counterstained with DAPI (blue). Merged images illustrate the spatial relationship between EMT-associated proteins, the actin cytoskeleton, and nuclei. Images were acquired using a 63× objective under identical imaging conditions. Scale bars: 10 µm.

To further examine EMT-associated protein levels and subcellular distribution, confocal IF staining was performed (Figure 6). Under identical imaging conditions, LLGL1 KO cells displayed reduced ZEB1 immunoreactivity compared with control cells, with weaker nuclear-localized signals (Figure 6A). In contrast, E-Cadherin staining intensity was increased in LLGL1 KO cells and showed enhanced peripheral localization (Figure 6B). Vimentin immunoreactivity, which was robust in control cells, was markedly reduced in LLGL1 KO cells (Figure 6C). Phalloidin staining revealed comparable overall F-actin organization between control and LLGL1 KO cells (Figure 6). These observations document altered protein abundance and subcellular distribution of EMT-associated markers in LLGL1 KO Huh-7 cells.

**Figure 7:**
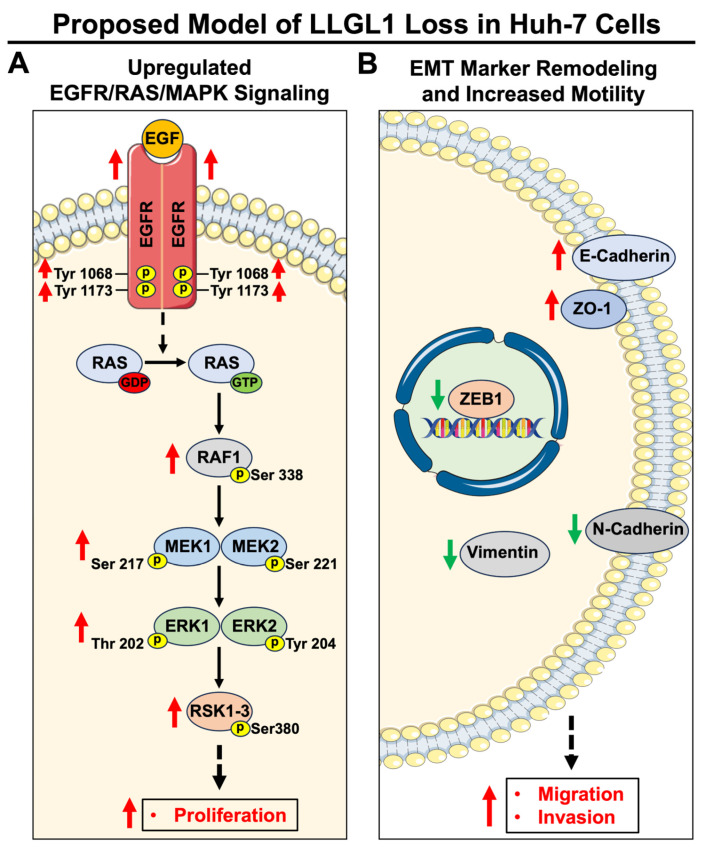
Model summarizing the effects of LLGL1 loss in Huh-7 cells. Schematic summary of the major signaling, molecular, and phenotypic changes observed following LLGL1 loss in Huh-7 cells, summarizing experimental findings on EGFR/MAPK pathway activity, EMT-associated marker expression, and cellular behaviors. (**A**) LLGL1 ablation enhances EGFR/RAS/MAPK signaling upon EGF stimulation, reflected by increased phosphorylation of EGFR at Tyr1068 and Tyr1173 and downstream activation of the RAF1-MEK1/2-ERK1/2-RSK1-3 cascade. These signaling alterations are associated with increased proliferative output observed in LLGL1 KO cells. (**B**) LLGL1 loss is accompanied by remodeling of EMT-associated marker protein levels, including increased E-Cadherin and ZO-1, reduced mesenchymal markers (N-Cadherin and Vimentin), and reduced nuclear ZEB1, together with increased cell motility. Red arrows indicate relative increases, green arrows indicate relative decreases, and dashed arrows indicate indirect or downstream relationships. GDP: Guanosine diphosphate; GTP: Guanosine triphosphate; p: phosphorylation. Adapted from Servier Medical Art (https://smart.servier.com accessed on 28 February 2026), licensed under CC BY 4.0 (https://creativecommons.org/licenses/by/4.0/ accessed on 28 February 2026).

Finally, a schematic model is provided to summarize the major signaling and phenotypic changes identified upon LLGL1 loss in Huh-7 cells (Figure 7). LLGL1 ablation was associated with enhanced EGFR/RAS/MAPK pathway activation in response to EGF stimulation, as reflected by increased EGFR phosphorylation and downstream activation of the RAF1–MEK–ERK–RSK signaling cascade (Figure 7A). In parallel, LLGL1 loss was associated with remodeling of EMT-associated marker protein levels without evidence of a classical EMT program, together with increased migratory and invasive activity (Figure 7B). This schematic summarizes coordinated experimental observations. Direct MAPK dependence of the EMT-associated marker changes was not tested in this study.

## 3. Discussion

Using LLGL1 KO and LLGL1-expressing control Huh-7 HCC cells, this study describes the functional contribution of LLGL1 to HCC-associated cellular phenotypes. LLGL1 loss confers a proliferative advantage, reflected by accelerated population expansion and increased clonogenic capacity (Figure 2A,C). Consistent with these findings, this growth-promoting effect is accompanied by a shift in cell-cycle distribution toward S-phase enrichment without alteration of the G2/M fraction (Figure 2B), which is compatible with a role for LLGL1 in restraining cell-cycle progression. Taken together, these results support a role for LLGL1 as a negative regulator of proliferative behavior in HCC cells.

Analysis of EGFR pathway dynamics revealed that LLGL1 ablation is associated with relief of a regulatory constraint on mitogenic signaling, leading to enhanced EGFR/RAS/MAPK pathway activity (Figure 3). A striking observation was the increase in total EGFR protein abundance in LLGL1 KO cells, accompanied by marked hyperphosphorylation at both Tyr1068 and Tyr1173 (Figure 3A–D). Phosphorylation of EGFR at these residues recruits adaptor complexes that link the receptor to downstream RAS/MAPK signaling [20]. Concurrent activation of both sites therefore reflects a broad enhancement of EGFR signaling capacity rather than preferential engagement of a single pathway branch. In line with prior work demonstrating that LLGL1 restrains EGFR-dependent migratory and survival programs by limiting receptor-associated signaling platforms at the plasma membrane [13], our data extend this concept to HCC by showing that LLGL1 loss is associated with increased EGFR abundance and phosphorylation, together with enhanced activation of canonical downstream signaling cascades. To further support this interpretation, a non-permeabilized IF analysis was included to qualitatively assess surface-associated EGFR, providing evidence that increased total EGFR levels in LLGL1 KO cells are accompanied by enhanced membrane-localized receptor availability. Notably, this regulatory interaction appears to be bidirectional. Acute EGF stimulation was associated with a reduction in LLGL1 protein abundance in control cells (Figure 3A). Given the short duration of EGF exposure, this effect is more consistent with altered protein stability than with transcriptional regulation, suggesting EGFR-driven turnover of LLGL1. In support of this interpretation, EGF has previously been shown to suppress the related polarity protein LLGL2 in primary hepatocytes [21], indicating that EGFR signaling can negatively regulate Lgl-family components to facilitate rapid remodeling of epithelial polarity programs. Together, these findings support a reciprocal regulatory association between LLGL1 and EGFR signaling. Loss of LLGL1 is associated with enhanced EGFR signaling output, whereas EGFR activation, in turn, is associated with reduced LLGL1 protein abundance.

In line with these upstream signaling changes, EGF stimulation led to a pronounced reduction in total RAF1 protein levels in control cells, whereas RAF1 abundance was relatively preserved in LLGL1 KO cells (Figure 3A,F,G). Given the short stimulation window, this effect is unlikely to reflect transcriptional regulation and instead is more consistent with post-translational control of RAF1 protein stability. As RAF1 turnover is tightly regulated through ubiquitin–proteasome-dependent mechanisms [22], these data are consistent with the possibility that LLGL1 status influences RAF1 stability during EGFR-driven signaling.

Western blotting analyses further indicated coordinated phosphorylation of MEK1/2, ERK1/2, and RSK1–3 downstream of EGFR activation (Figure 3A,F–J). Together, these changes are consistent with robust MAPK signaling output in LLGL1 KO cells. As RSK functions as a key effector linking ERK signaling to transcriptional and translational programs governing proliferation and survival [23], elevated RSK activation may contribute to the S-phase enrichment and increased clonogenic capacity observed upon LLGL1 loss (Figure 2). Further studies will be required to directly define MAPK-dependent transcriptional programs downstream of RSK activation in this context.

Together, these findings support the concept of LLGL1 as an epithelial signaling brake that constrains EGFR-driven RAS/MAPK pathway propagation at multiple levels. Although the present study relies on a single LLGL1 KO clone, LLGL1 ablation was achieved using a paired-guide CRISPR/Cas9n strategy, which is known to reduce off-target editing [24]. Complete loss of LLGL1 protein was confirmed at the protein level (Figure 3 and Figure 5). In our signaling experiments, pharmacological EGFR inhibition attenuated EGFR and downstream MAPK phosphorylation events, supporting effective pathway blockade at the biochemical level (Figure 3). However, we did not directly test whether EGFR or MEK inhibition reverses the enhanced migration and invasion phenotypes or the remodeling of EMT-associated marker proteins. Accordingly, the observed functional effects are framed as being associated with, rather than causally dependent on, EGFR/RAS/MAPK signaling in this study. Importantly, we do not claim a direct causal relationship between MAPK output and EMT-associated marker remodeling. Instead, the observed phenotypes were mutually consistent, including enhanced proliferation, increased EGFR/RAS/MAPK signaling activity, and elevated migration and invasion. These effects are in line with the established tumor-suppressive functions of LLGL1 across epithelial systems [7,8]. Rather than broadly increasing pathway component expression, LLGL1 loss selectively enhanced receptor abundance and signaling efficiency, resulting in coordinated amplification of mitogenic signaling. This regulatory mode aligns with emerging concepts in which epithelial polarity proteins shape oncogenic signaling by organizing spatial signaling domains, rather than acting as simple binary suppressors [25,26,27]. In HCC, where MAPK pathway activation frequently reflects upstream regulatory deregulation rather than recurrent RAS mutations [28,29], our data highlight loss of LLGL1 as a previously underappreciated mechanism through which epithelial disorganization may potentiate growth-promoting signaling. Despite the use of a paired-guide Cas9n strategy and genetic validation to minimize off-target concerns, future studies incorporating LLGL1 re-expression approaches will be required to strengthen causal interpretation. In addition, extending the analysis of LLGL1 loss to additional HCC cell lines will be important to assess the broader relevance of these findings across heterogeneous tumor contexts. Accordingly, all functional and mechanistic conclusions in the present study are derived from Huh-7 cells. In light of the known heterogeneity of HCC and the context-dependent reliance on EGFR/MAPK signaling across experimental models, the observed phenotypic and signaling alterations primarily reflect the Huh-7 context [30]. Evaluation of these phenotypes in additional HCC cell lines representing distinct epithelial and mesenchymal states will be essential to further assess generalizability.

Huh-7 cells originate from a well-differentiated HCC and are commonly categorized as a well-differentiated, epithelial-like HCC model in comparative liver cancer cell line panels [31,32]. Consistently, integrative multi-omic profiling studies place Huh-7 within a global epithelial-like state, supporting its suitability for interrogating epithelial polarity-linked signaling and EMT/EMP-associated marker remodeling [33]. Moreover, Huh-7 cells are widely used to study EGF/EGFR-driven signaling outputs, including MAPK pathway activation and growth factor-induced remodeling of EMT-associated marker expression in HCC models [34,35]. Together, these features support the use of Huh-7 as a rational epithelial-like HCC platform to interrogate polarity-linked EGFR/MAPK signaling and EMT-associated marker remodeling in the present study.

Results of the migration and invasion assays revealed that LLGL1 loss markedly enhances migratory and invasive behavior of Huh-7 cells (Figure 4), extending to HCC the broader concept that Scribble-module polarity components function as epithelial restraints on motility programs. Consistent with this view, reduced LLGL1 expression or mislocalization has been repeatedly associated with increased invasiveness across multiple epithelial tumor systems, supporting a conserved anti-motility role for LLGL1 in maintaining epithelial organization [7,8,9,11,36]. Mechanistically, prior work has shown that LLGL1 constrains EGF-dependent migratory and survival programs, reinforcing the concept that polarity proteins regulate growth factor-driven cell-state transitions rather than merely stabilizing static epithelial architecture [13]. In the context of HCC, where invasive dissemination critically influences recurrence and outcome, these findings are compatible with emerging models in which polarity loss contributes to signaling reprogramming that licenses motility and invasion [1,2].

A central finding of this study is that the enhanced migration and invasion observed in LLGL1 KO Huh-7 cells occurred in the absence of a canonical “full EMT” marker pattern, as assessed in this study (Figure 5 and Figure 6). Importantly, the absence of a “full EMT” refers specifically to the lack of a classical junction-to-mesenchymal marker switch within the marker panel analyzed (Figure 5 and Figure 6), rather than exclusion of broader EMT- or EMP-related programs. Consistent with contemporary frameworks, increased motility may arise through EMP and collective migration states that retain, or even strengthen, junctional proteins while rewiring polarity, adhesion turnover, and cytoskeleton–junction coupling. Within this context, elevated E-Cadherin and ZO-1 may support coordinated movement, whereas reduced Vimentin, N-Cadherin, and nuclear ZEB1 suggest that LLGL1 loss does not drive a stable mesenchymal conversion in Huh-7 cells. Instead, LLGL1 loss was associated with an epithelial-weighted remodeling of EMT-associated marker expression, characterized by maintenance or upregulation of junctional epithelial proteins alongside reduction of mesenchymal markers. Such a phenotype is not paradoxical within current EMT paradigms, as EMT is now widely recognized as a continuum of intermediate or hybrid epithelial–mesenchymal states that can support invasion and dissemination without complete lineage conversion [37,38,39]. In HCC, where EMT is difficult to capture as a binary state in patient material, invasive competence is often more accurately explained by EMP and spatially localized invasion-associated programs than by stable mesenchymal conversion [40]. Notably, such hybrid states can equal or exceed fully mesenchymal phenotypes in aggressiveness, as they preserve multicellular fitness advantages while acquiring motility and invasion modules [37,39,41]. Consistently, the coexistence of reinforced epithelial junctional markers with increased migration and invasion is fully compatible with contemporary EMT frameworks, which recognize EMP as a spectrum rather than a binary transition [37,42]. Within this spectrum, hybrid or epithelial-weighted EMP states can retain, or even upregulate, junctional components such as E-Cadherin and ZO-1 while simultaneously activating motility and invasion programs [43]. In these contexts, E-Cadherin-based cell–cell adhesion can facilitate coordinated or collective migration by preserving multicellular cohesion, rather than acting solely as a static barrier to movement [44,45]. Accordingly, the epithelial-weighted remodeling of EMT-associated markers observed upon LLGL1 loss is fully compatible with the increased migratory and invasive phenotypes identified in Huh-7 cells, without implying a classical or complete EMT program.

A key point is that increased migration and invasion do not necessarily require a complete loss of epithelial junctional proteins. Hybrid epithelial–mesenchymal states can retain or even reinforce E-Cadherin-based adhesion while activating invasive machinery and collective migration programs [46]. In this setting, E-Cadherin may support coordinated movement and may participate in pro-invasive structures such as invadopodia, providing a plausible mechanistic explanation for enhanced transwell motility despite an epithelial-weighted marker profile [47]. Recent work supports the concept that E-Cadherin–positive hybrid states can be pro-invasive and that EMT is a dynamic, context-dependent continuum rather than a binary switch [48]. Additionally, a broader EMT/EMP marker panel (such as SLUG/TWIST family factors, EpCAM/cytokeratins, fibronectin, and integrin programs) was not assessed here and will be valuable to include in future studies to further resolve the molecular basis of this epithelial-weighted motile state.

IF analyses support the biochemical data by confirming that changes observed by immunoblotting for ZEB1, E-Cadherin, and Vimentin are reflected at the single-cell level, while also revealing spatial features relevant to the migratory phenotype associated with LLGL1 loss (Figure 5 and Figure 6). Altered subcellular distribution of polarity- and junction-associated proteins suggests that LLGL1 ablation perturbs epithelial spatial organization in a manner that may influence signaling–cytoskeleton coupling [1,2]. Notably, phalloidin staining revealed changes in cortical actin patterning without evidence of widespread cytoskeletal collapse, indicating a reorganization of actin architecture rather than a loss of structural integrity. Such remodeling is consistent with a migratory state driven by altered junctional coordination rather than wholesale cytoskeletal reprogramming [49]. Within this framework, LLGL1 loss may relax junction–cortex coupling and epithelial spatial constraints while EGFR–MAPK signaling is concurrently amplified. Together, these observations provide a plausible biological context for increased migration and invasion in LLGL1 KO Huh-7 cells, alongside the amplified EGFR–MAPK signaling state.

Beyond EMT-associated mechanisms, polarity-sensitive signaling pathways may also contribute to the migratory and invasive phenotypes observed upon LLGL1 loss. The Hippo–YAP/TAZ pathway represents a central polarity-responsive signaling axis implicated in HCC progression and invasion [50,51,52,53]. Consistent with this framework, emerging evidence suggests that LLGL1 may influence Hippo pathway output at the level of YAP/TAZ stability or subcellular localization, providing a potential mechanistic link between polarity loss and pro-migratory transcriptional programs [13,54]. In addition, integrin–FAK signaling is a well-established driver of adhesion turnover and invasion in HCC [55,56]. Given LLGL1′s role in organizing epithelial polarity and junctional architecture, future studies will be required to determine whether LLGL1 loss modulates these pathways independently or in coordination with EGFR/RAS/MAPK signaling.

In summary, our findings highlight LLGL1 as an epithelial polarity regulator that shapes how HCC cells interpret growth factor-driven oncogenic signaling. Loss of LLGL1 is associated with enhanced EGFR/RAS/MAPK pathway output, increased proliferative capacity, and elevated migration and invasion through epithelial plasticity rather than classical EMT. By positioning LLGL1 at the intersection of epithelial organization, signal transduction, and invasive behavior, this study provides a conceptual framework linking polarity loss to HCC biology and underscores epithelial polarity control as a functionally relevant dimension of tumor biology.

## 4. Materials and Methods

### 4.1. Cell Culture

The human HCC cell line Huh-7 was used in all experiments. Huh-7 cells were originally established from a well-differentiated human HCC, as described in the original report characterizing this cell line [57]. Dulbecco’s modified Eagle’s medium (DMEM) (Catalog Number (Cat. No): 41966029; Thermo Fisher Scientific, Waltham, MA, USA) supplemented with 10% fetal bovine serum (FBS) (Cat. No: A5256801; Thermo Fisher Scientific, Waltham, MA, USA), 1% penicillin-streptomycin (Cat. No: 15140122; Thermo Fisher Scientific, Waltham, MA, USA), and non-essential amino acids (Cat. No: 11140050; Thermo Fisher Scientific, Waltham, MA, USA), was used as the complete culture medium for maintaining the Huh-7 cell line. Cells were cultured at 37 °C in a humidified atmosphere of 5% CO_2_ and grown as monolayers. Cells were detached from culture plates using trypsin-EDTA solution (Cat. No: 25200056; Thermo Fisher Scientific, Waltham, MA, USA) for passaging and experimental procedures. Antibiotic-free freezing medium containing 20% FBS and 10% dimethyl sulfoxide (DMSO) (Cat. No: D8418; Sigma-Aldrich, St. Louis, MO, USA) in DMEM was used for cryopreservation of cells in liquid nitrogen. Serum starvation was performed by culturing cells in FBS-free medium for 24 h prior to stimulation. EGFR signaling was inhibited by pre-treating cells with 10 μM gefitinib (Cat. No: S1025; Selleck Chemicals, Houston, TX, USA) for 1 h prior to stimulation with 100 ng/mL recombinant EGF (Cat. No: PHG0313; Thermo Fisher Scientific, Waltham, MA, USA) for 15 min. Short tandem repeat (STR) profiling was not performed in the present study. However, routine cell culture practices were followed, and all cultures were confirmed to be mycoplasma-free prior to experimentation (Mycoplasma PCR Detection Kit, Cat. No: G238; abm, Richmond, BC, Canada).

### 4.2. CRISPR/Cas9n Plasmid Design and Genome Editing of Huh-7 Genome

*LLGL1* KO Huh-7 cells were generated using the CRISPR/Cas9n system [24]. To identify target sites for genome editing and design sgRNA sequences, the human *LLGL1* genomic sequence was analyzed using CCTop and CRISPOR online tools [58,59]. Two target sites in the first exon of *LLGL1* were selected. Oligonucleotides containing BbSI restriction enzyme recognition sites were ordered (Macrogen, Seoul, South Korea) for cloning into the PX462 expression vector (plasmid No: 62987; Addgene, Watertown, MA, USA). The oligonucleotides used to construct sgRNAs targeting the human *LLGL1* gene were as follows: sgRNA-1F (5′-caccGGAGCTTTTCGCCTTCAACA-3′), sgRNA-1R (5′-aaacTGTTGAAGGCGAAAAGCTCC-3′), sgRNA-2F (5′-caccgTGCTTGAGCTTCTCGCGCTG-3′), and sgRNA-2R (5′-aaacCAGCGCGAGAAGCTCAAGCAc-3′). Scrambled control (SC) sgRNA sequences, previously described as negative control guides [60], were also purchased and used. The oligonucleotides used to construct SC gRNAs were: SCsgRNA-F (5′-caccgTATTACTGATATTGGTGGG-3′) and SCsgRNA-R (5′-aaacCCCACCAATATCAGTAATAc-3′). Double-stranded gRNA oligonucleotides were generated by annealing complementary sgRNA pairs with T4 polynucleotide kinase (Cat. No: M2622; New England Biolabs, Ipswich, MA, USA) according to the manufacturer’s instructions. The annealed oligonucleotide pairs and the PX462 vector were digested with BbSI (Cat. No: ER1011; Thermo Fisher Scientific, Waltham, MA, USA) and subsequently ligated to generate sgRNA-expressing constructs. Constructs that contained LLGL1 sgRNA-encoding sequences were designated as PX462-LLGL1-1 and PX462-LLGL1-2, whereas SC sgRNA-encoding plasmid was designated as PX462-SC.

To generate *LLGL1* KO and negative control Huh-7 clones, 1.5 × 10^6^ Huh-7 cells were co-transfected with 1.25 μg each of PX462-LLGL1-1 and PX462-LLGL1-2 or 2.5 μg of PX462-SC using FuGENE HD transfection reagent (Cat. No: E2311; Promega, Madison, WI, USA). After 24 h of culture, transfected cells were trypsinized and seeded into 96-well plates as single colonies (1 cell/well). Twelve hours later, 1.75 μg/mL of puromycin (Cat. No: A11138; Thermo Fisher Scientific, Waltham, MA, USA) was applied for selection. After 72 h, puromycin selection was discontinued by replacing the medium with puromycin-free culture medium. One week later, colonies were transferred to 24-well plates. Following that, the expanded colonies were transferred into 12-well plates. After two weeks of culturing, the cells were trypsinized, split into two fractions; one for culturing and one for the analyses of LLGL1 protein levels. A LLGL1 KO Huh-7 clone lacking detectable LLGL1 protein expression and a LLGL1-expressing control Huh-7 clone were selected for subsequent experiments.

### 4.3. Sanger DNA Sequencing

gRNA-encoding sequences cloned into PX462 vectors and genomic *LLGL1* loci from selected LLGL1 KO and SC CRISPR/Cas9n Huh-7 clones were analyzed by Sanger DNA sequencing using an ABI PRISM 3130 Genetic Analyzer (Applied Biosystems, Foster City, CA, USA). Prior to Sanger sequencing, genomic DNA from Huh-7 clones was isolated using the NucleoSpin DNA Isolation kit (Cat. No: 740952; Macherey-Nagel, Düren, Germany). The *LLGL1* target region was amplified by PCR using gene-specific primers (Forward: 5′-CCCCTCCAAAGCGCTTCCCCAG-3′ and Reverse: 5′-GAGACCCAGACCCACCCAGAGTCC-3′). PCR reactions were performed in a total volume of 25 µL containing 0.5 µL PrimeSTAR GXL DNA polymerase (Cat. No: R050Q; Takara Bio, Shiga, Japan), 5 µL 5× GXL buffer, 1 µL genomic DNA (100 ng/µL), 2 µL 2.5 mM dNTP mixture (Cat. No: U1420; Promega, Madison, WI, USA), 0.5 µL each of forward (F) and reverse (R) primers (10 pmol/µL), and 15.5 µL nuclease-free water. PCR cycling conditions were as follows: 2 min at 98 °C; 35 cycles of 10 s at 98 °C, 20 s at 67 °C, 45 s at 72 °C; followed by a final extension for 5 min at 72 °C and hold at 10 °C. PCR products were verified by gel electrophoresis and purified using the HiPure Gel Extraction Kit (Cat. No: D211102; Magen, Guangzhou, China). Sanger sequencing reactions were performed on purified LLGL1 PCR products using the same primers employed for PCR amplification. gRNA inserts cloned into PX462 vectors were also verified by Sanger sequencing using the same method and a vector-specific primer (5′-GAGGGCCTATTTCCCATGATTCC-3′). Sequencing reactions were performed using the Brilliant Dye Terminator Kit (Cat. No: BRD03-100, Nimagen, Nijmegen, The Netherlands) with a reaction mixture containing 0.8 µL Brilliant Dye, 1.7 µL 5× buffer, 0.5 µL primer, 7 µL DNA template, and nuclease-free water. Sequencing cycling conditions were as follows: 45 s at 96 °C; 28 cycles of 10 s at 96 °C, 5 s at 50 °C, 2 min at 60 °C; followed by a hold at 10 °C. Sequencing data were analyzed using SnapGene Viewer software version (v.7.2) and compared with reference human genome sequences.

### 4.4. Doubling Time (DT) Analysis

To determine the population DTs of LLGL1 KO and control Huh-7 cells, 3.0 *×* 10^5^ cells were seeded per well in 6-well plates in triplicate. The number of viable cells was determined at 24 h intervals for up to 72 h using a BD Accuri C6 flow cytometer and BD Accuri C6 software v.1.0.264 (BD Biosciences, San Jose, CA, USA) up to 72 h. Population DTs were calculated and growth curves were generated using GraphPad Prism software v.9.5 with the “*exponential growth with log(population)*” function.

### 4.5. Cell Cycle Analysis

Control and LLGL1 KO Huh-7 cells were seeded into 6-well plates and cultured for 48 h prior to cell cycle analysis. Cells were then trypsinized, counted using a flow cytometer, and 1.0 × 10^6^ cells per condition were fixed in 70% ethanol. Fixed cells were washed with phosphate-buffered saline (PBS) and incubated with PI/RNAse staining solution (Cat. No: 550825; BD Biosciences, San Jose, CA, USA) for 15 min at room temperature (RT) in the dark. Samples were analyzed using a BD Accuri C6 flow cytometer, and cell cycle distribution was quantified using ModFit software (v.4.1.7; BD Biosciences, San Jose, CA, USA). Data from three independent experiments were used for subsequent statistical analysis.

### 4.6. Colony Formation Assay

For the colony formation assay, 600 cells per well were seeded into 6-well plates and cultured for two weeks. Cells were then fixed with 4% paraformaldehyde (PFA) (Cat. No: P6148, Sigma-Aldrich, St. Louis, MO, USA), stained with 0.1% crystal violet (Cat. No: HT90132; Sigma-Aldrich, St. Louis, MO, USA), and washed with distilled water. Plates were air-dried and imaged. Colonies were quantified using ImageJ software v.1.54g. Three independent biological replicates were performed per condition. Colony numbers in LLGL1 KO cells were normalized to control cells and analyzed using GraphPad Prism software v.9.5.

### 4.7. Protein Extraction and Western Blot Analysis

Proteins were extracted from cells using TNTE lysis buffer (50 mM Tris-HCl, pH 7.4; 150 mM NaCl; 1 mM ethylenediaminetetraacetic acid (EDTA); 1% Triton X-100 (Cat. No: X-100; Sigma-Aldrich); 5 mM sodium pyrophosphate; 2 mM sodium orthovanadate; 20 mM sodium fluoride; 1 mM phenylmethylsulfonyl fluoride (PMSF); and 1× protease inhibitor cocktail tablet (Cat. No: S8830-20TAB; Sigma-Aldrich, St. Louis, MO, USA). Protein concentrations were measured using a bicinchoninic acid (BCA) protein assay kit (Cat. No: 23225; Thermo Fisher Scientific, Waltham, MA, USA). Thirty micrograms of protein from each sample were boiled in sodium dodecyl sulfate-polyacrylamide gel electrophoresis (SDS-PAGE) loading buffer (0.25 M Tris-Cl (pH 6.8), 10% SDS, 0.01% bromophenol blue) and resolved on 7.5% or 10% SDS-PAGE gels, depending on target protein size. Proteins were transferred onto nitrocellulose membranes (Cat. No: 1620115; Bio-Rad, Hercules, CA, USA). Membranes were blocked with 5% non-fat dry milk prepared in Tris-buffered saline containing 0.05% Tween-20 (TBS-T). Primary and secondary antibodies listed in Table S1 were applied according to the manufacturers’ instructions. Membranes were washed three times with TBS-T buffer after antibody incubations. Membranes were incubated with enhanced chemiluminescence (ECL) (Cat. No: 1705060; Bio-Rad, Hercules, CA, USA), and protein bands were visualized with the ChemiDoc MP Imaging System (Bio-Rad). When required, membranes were incubated with acetic acid and reprobed with primary antibodies raised in a different host species to enable detection of additional targets on the same membrane [61]. For quantitative analysis of protein levels, densitometric analysis of protein band intensities was performed using Image Lab software v.6.1.0 (Bio-Rad, Hercules, CA, USA). Target protein signals were normalized to the corresponding Beta Actin band intensity to account for variations in protein loading and transfer efficiency. The normalized values were subsequently scaled to the respective control group, which was defined as 1, to calculate relative protein expression levels. For selected phosphorylation readouts, densitometric values were log_10_-transformed prior to statistical analysis to improve data distribution and variance homogeneity. These relative values were used for all downstream graphical representations and statistical analyses. For EMT-associated Western blot analyses, protein lysates derived from three independent biological samples collected at different time points were resolved and analyzed on the same gel and membrane to minimize technical variability.

### 4.8. Transwell Migration and Invasion Assays

Cell migration and invasion assays were performed using transwell inserts (Boyden chambers) with 8.0 µm pore size membranes for 24-well plates (Cat. No. 353097; Corning, NY, USA). For migration assays, cells were serum-starved in serum-free DMEM for 24 h, then trypsinized and resuspended in serum-free medium. A total of 5.0 × 10^4^ cells per insert, suspended in 0.4 mL of serum-free DMEM, were seeded into the upper chambers in triplicate. The lower chambers were filled with 0.6 mL of DMEM containing 10% FBS as a chemoattractant. For invasion assays, transwell inserts were pre-coated with Matrigel (Cat. No: 356234; Sigma-Aldrich, St. Louis, MO, USA) prior to cell seeding. Following incubation for 48 h and 72 h for migration assays, and 72 h for invasion assays, cells on the upper surface of the membrane were removed using a cotton swab. Migrated or invaded cells on the lower membrane surface were fixed with 4% PFA for 20 min, stained with 0.1% crystal violet for 20 min, and washed three times with distilled water. Five randomly selected microscopic fields per transwell insert were imaged and migrated or invaded cells were quantified using ImageJ software.

### 4.9. IF Staining and Confocal Microscopy

For IF staining of EMT-associated proteins, LLGL1 KO and control Huh-7 cells were cultured on glass coverslips in 6-well plates in triplicate with complete DMEM. After 24 h, culture medium was removed, cells were washed three times with PBS, and fixed with 4% PFA in PBS for 15 min at RT. Cells were permeabilized with 0.2% Triton X-100 in PBS for 10 min at RT, and subsequently blocked with 5% bovine serum albumin (BSA; Cat. No: BSA-1S; Capricorn Scientific, Ebsdorfergrund, Germany) for 1 h at RT. Coverslips were incubated overnight at 4 °C with primary antibodies listed in Table S1. After incubation, coverslips were washed three times with PBS. Cells were incubated with Phalloidin iFluor 488 reagent (Cat. No: ab176753, Abcam, Cambridge, UK) for 30 min at RT to stain F-actin, washed with PBS, and then incubated with far-red fluorophore-conjugated secondary antibodies for 1 h at RT. For assessment of surface-associated EGFR, IF staining was performed under non-permeabilized conditions by omitting the Triton X-100 permeabilization step. Following antibody incubations, slides were washed with PBS and mounted using ProLong Gold Anti-fade Reagent containing 4′,6-diamidino-2-phenylindole (DAPI) (Cat. No: P36935; Thermo Fisher Scientific, Waltham, MA, USA). For IF experiments, three independently prepared biological samples per condition were stained and processed simultaneously to ensure identical experimental conditions. Slides were imaged using a Leica SP8 laser-scanning confocal microscope equipped with a 63× objective, and images were processed using LAS X software v.3.5.5.

### 4.10. Statistical Analyses

Statistical analyses were performed using GraphPad Prism software v.9.5. The data were obtained from three independent biological experiments and are expressed as mean ± standard error of the mean (SEM) in graphs. Cell number data obtained from growth curve experiments were analyzed using multiple unpaired *t*-tests with Holm–Šidák correction for multiple comparisons. DTs were calculated from the corresponding growth curves. Colony formation, migration, and invasion data were analyzed using unpaired two-tailed *t*-tests. Cell cycle distribution data were analyzed using unpaired two-tailed *t*-tests with Welch’s correction. Data from EGFR/RAS/MAPK Western blotting experiments were analyzed using one-way ANOVA followed by Tukey’s multiple-comparisons post hoc test, with log_10_ transformation applied to selected datasets as specified. Western blotting data for EMT-associated markers were analyzed using unpaired two-tailed *t*-tests. In all analyses, a *p*-value < 0.05 was considered statistically significant. Statistical significance is indicated in graphs as follows: * *p* < 0.05, ** *p* < 0.01, *** *p* < 0.001, and **** *p* < 0.0001.

## 5. Conclusions

In conclusion, our findings identify LLGL1 as an epithelial polarity regulator that modulates growth factor-driven oncogenic signaling responses in Huh-7 cells. LLGL1 loss promotes increased EGFR/RAS/MAPK signaling activity, enhanced proliferation, and elevated migratory and invasive behavior in Huh-7 cells. These phenotypic changes occur in the context of epithelial plasticity rather than a classical EMT transition. Overall, our results define an LLGL1-dependent signaling and phenotypic program in Huh-7 cells and offer a framework for future evaluation across additional HCC models to assess broader applicability.

## Data Availability

The original contributions presented in this study are included in the article/Supplementary Materials. Further inquiries can be directed to the corresponding author.

## Supplementary Materials

The supporting information can be downloaded at: https://www.mdpi.com/article/10.3390/ijms27072959/s1.

## References

[1] Buckley C.E., St Johnston D. (2022). Apical-basal polarity and the control of epithelial form and function. Nat. Rev. Mol. Cell Biol..

[2] McCaffrey L.M., Macara I.G. (2011). Epithelial organization, cell polarity and tumorigenesis. Trends Cell Biol..

[3] Hanahan D. (2022). Hallmarks of Cancer: New Dimensions. Cancer Discov..

[4] Grifoni D., Garoia F., Schimanski C.C., Schmitz G., Laurenti E., Galle P.R., Pession A., Cavicchi S., Strand D. (2004). The human protein Hugl-1 substitutes for *Drosophila* lethal giant larvae tumour suppressor function in vivo. Oncogene.

[5] Yamanaka T., Horikoshi Y., Sugiyama Y., Ishiyama C., Suzuki A., Hirose T., Iwamatsu A., Shinohara A., Ohno S. (2003). Mammalian Lgl forms a protein complex with PAR-6 and aPKC independently of PAR-3 to regulate epithelial cell polarity. Curr. Biol..

[6] Yamanaka T., Horikoshi Y., Izumi N., Suzuki A., Mizuno K., Ohno S. (2006). Lgl mediates apical domain disassembly by suppressing the PAR-3-aPKC-PAR-6 complex to orient apical membrane polarity. J. Cell Sci..

[7] Schimanski C.C., Schmitz G., Kashyap A., Bosserhoff A.K., Bataille F., Schafer S.C., Lehr H.A., Berger M.R., Galle P.R., Strand S. (2005). Reduced expression of Hugl-1, the human homologue of *Drosophila* tumour suppressor gene lgl, contributes to progression of colorectal cancer. Oncogene.

[8] Kuphal S., Wallner S., Schimanski C.C., Bataille F., Hofer P., Strand S., Strand D., Bosserhoff A.K. (2006). Expression of Hugl-1 is strongly reduced in malignant melanoma. Oncogene.

[9] Biesterfeld S., Kauhausen A., Kost C., Gockel I., Schimanski C.C., Galle P.R. (2012). Preservation of HUGL-1 expression as a favourable prognostic factor in pancreatic carcinoma. Anticancer Res..

[10] Matsuzaki T., Takekoshi S., Toriumi K., Kitatani K., Nitou M., Imamura N., Ogura G., Masuda R., Nakamura N., Iwazaki M. (2015). Reduced Expression of Hugl 1 Contributes to the Progression of Lung Squamous Cell Carcinoma. Tokai J. Exp. Clin. Med..

[11] Gont A., Hanson J.E., Lavictoire S.J., Daneshmand M., Nicholas G., Woulfe J., Kassam A., Da Silva V.F., Lorimer I.A. (2014). Inhibition of glioblastoma malignancy by Lgl1. Oncotarget.

[12] Lavoie H., Gagnon J., Therrien M. (2020). ERK signalling: A master regulator of cell behaviour, life and fate. Nat. Rev. Mol. Cell Biol..

[13] Greenwood E., Maisel S., Ebertz D., Russ A., Pandey R., Schroeder J. (2016). Llgl1 prevents metaplastic survival driven by epidermal growth factor dependent migration. Oncotarget.

[14] Zhu Y.X., Li C.H., Li G., Feng H., Xia T., Wong C.H., Fung F.K.C., Tong J.H., To K.F., Chen R. (2020). LLGL1 Regulates Gemcitabine Resistance by Modulating the ERK-SP1-OSMR Pathway in Pancreatic Ductal Adenocarcinoma. Cell Mol. Gastroenterol. Hepatol..

[15] Lu X., Feng X., Man X., Yang G., Tang L., Du D., Zhang F., Yuan H., Huang Q., Zhang Z. (2009). Aberrant splicing of Hugl-1 is associated with hepatocellular carcinoma progression. Clin. Cancer Res..

[16] Bray F., Laversanne M., Sung H., Ferlay J., Siegel R.L., Soerjomataram I., Jemal A. (2024). Global cancer statistics 2022: GLOBOCAN estimates of incidence and mortality worldwide for 36 cancers in 185 countries. CA Cancer J. Clin..

[17] Llovet J.M., Kelley R.K., Villanueva A., Singal A.G., Pikarsky E., Roayaie S., Lencioni R., Koike K., Zucman-Rossi J., Finn R.S. (2021). Hepatocellular carcinoma. Nat. Rev. Dis. Primers.

[18] Wang Y., Deng B. (2023). Hepatocellular carcinoma: Molecular mechanism, targeted therapy, and biomarkers. Cancer Metastasis Rev..

[19] Xue Y., Ruan Y., Wang Y., Xiao P., Xu J. (2024). Signaling pathways in liver cancer: Pathogenesis and targeted therapy. Mol. Biomed..

[20] Salazar-Cavazos E., Nitta C.F., Mitra E.D., Wilson B.S., Lidke K.A., Hlavacek W.S., Lidke D.S. (2020). Multisite EGFR phosphorylation is regulated by adaptor protein abundances and dimer lifetimes. Mol. Biol. Cell.

[21] Zimmermann T., Kashyap A., Hartmann U., Otto G., Galle P.R., Strand S., Strand D. (2008). Cloning and characterization of the promoter of Hugl-2, the human homologue of Drosophila lethal giant larvae (lgl) polarity gene. Biochem. Biophys. Res. Commun..

[22] Lu Z., Hunter T. (2009). Degradation of activated protein kinases by ubiquitination. Annu. Rev. Biochem..

[23] Wright E.B., Lannigan D.A. (2023). Therapeutic targeting of p90 ribosomal S6 kinase. Front. Cell Dev. Biol..

[24] Ran F.A., Hsu P.D., Lin C.Y., Gootenberg J.S., Konermann S., Trevino A.E., Scott D.A., Inoue A., Matoba S., Zhang Y. (2013). Double nicking by RNA-guided CRISPR Cas9 for enhanced genome editing specificity. Cell.

[25] Peglion F., Etienne-Manneville S. (2024). Cell polarity changes in cancer initiation and progression. J. Cell Biol..

[26] Casaletto J.B., McClatchey A.I. (2012). Spatial regulation of receptor tyrosine kinases in development and cancer. Nat. Rev. Cancer.

[27] Muthuswamy S.K., Xue B. (2012). Cell polarity as a regulator of cancer cell behavior plasticity. Annu. Rev. Cell Dev. Biol..

[28] Moon H., Ro S.W. (2021). MAPK/ERK Signaling Pathway in Hepatocellular Carcinoma. Cancers.

[29] Wheeler D.A., Roberts L.R. (2017). The Cancer Genome Atlas Research Network. Comprehensive and Integrative Genomic Characterization of Hepatocellular Carcinoma. Cell.

[30] Safri F., Nguyen R., Zerehpooshnesfchi S., George J., Qiao L. (2024). Heterogeneity of hepatocellular carcinoma: From mechanisms to clinical implications. Cancer Gene Ther..

[31] Rodriguez-Hernandez M.A., Chapresto-Garzon R., Cadenas M., Navarro-Villaran E., Negrete M., Gomez-Bravo M.A., Victor V.M., Padillo F.J., Muntane J. (2020). Differential effectiveness of tyrosine kinase inhibitors in 2D/3D culture according to cell differentiation, p53 status and mitochondrial respiration in liver cancer cells. Cell Death Dis..

[32] Vinh Hanh N., Thi Thanh Thuy L., Ngoc Hieu V., Hai H., Ikenaga H., Sato-Matsubara M., Uchida-Kobayashi S., Urushima H., Van Khanh N., Thi Ha N. (2024). Poorly Differentiated Hepatocellular Carcinoma Cells Avoid Apoptosis by Interacting with T Cells via CD40-CD40 Ligand Linkage. Am. J. Pathol..

[33] Wang S., Xie J., Zou X., Pan T., Yu Q., Zhuang Z., Zhong Y., Zhao X., Wang Z., Li R. (2022). Single-cell multiomics reveals heterogeneous cell states linked to metastatic potential in liver cancer cell lines. iScience.

[34] Niu J., Li W., Liang C., Wang X., Yao X., Yang R.H., Zhang Z.S., Liu H.F., Liu F.Y., Pei S.H. (2020). EGF promotes DKK1 transcription in hepatocellular carcinoma by enhancing the phosphorylation and acetylation of histone H3. Sci. Signal..

[35] Bertoldi A., Cusumano G., Calzoni E., Alabed H.B.R., Pellegrino R.M., Buratta S., Urbanelli L., Emiliani C. (2025). Multi-Omic Characterization of Epithelial-Mesenchymal Transition: Lipidomic and Metabolomic Profiles as Key Markers of TGF-beta-Induced Transition in Huh7 Hepatocellular Carcinoma. Cells.

[36] Russ A., Louderbough J.M., Zarnescu D., Schroeder J.A. (2012). Hugl1 and Hugl2 in mammary epithelial cells: Polarity, proliferation, and differentiation. PLoS ONE.

[37] Fontana R., Mestre-Farrera A., Yang J. (2024). Update on Epithelial-Mesenchymal Plasticity in Cancer Progression. Annu. Rev. Pathol..

[38] Malagoli Tagliazucchi G., Wiecek A.J., Withnell E., Secrier M. (2023). Genomic and microenvironmental heterogeneity shaping epithelial-to-mesenchymal trajectories in cancer. Nat. Commun..

[39] Zhang C.X., Huang R.Y., Sheng G., Thiery J.P. (2025). Epithelial-mesenchymal transition. Cell.

[40] Wu L., Yan J., Bai Y., Chen F., Zou X., Xu J., Huang A., Hou L., Zhong Y., Jing Z. (2023). An invasive zone in human liver cancer identified by Stereo-seq promotes hepatocyte-tumor cell crosstalk, local immunosuppression and tumor progression. Cell Res..

[41] Youssef K.K., Narwade N., Arcas A., Marquez-Galera A., Jimenez-Castano R., Lopez-Blau C., Fazilaty H., Garcia-Gutierrez D., Cano A., Galceran J. (2024). Two distinct epithelial-to-mesenchymal transition programs control invasion and inflammation in segregated tumor cell populations. Nat. Cancer.

[42] Lu W., Kang Y. (2019). Epithelial-Mesenchymal Plasticity in Cancer Progression and Metastasis. Dev. Cell.

[43] Aggarwal V., Montoya C.A., Donnenberg V.S., Sant S. (2021). Interplay between tumor microenvironment and partial EMT as the driver of tumor progression. iScience.

[44] Na T.Y., Schecterson L., Mendonsa A.M., Gumbiner B.M. (2020). The functional activity of E-cadherin controls tumor cell metastasis at multiple steps. Proc. Natl. Acad. Sci. USA.

[45] Rubtsova S.N., Zhitnyak I.Y., Gloushankova N.A. (2022). Dual role of E-cadherin in cancer cells. Tissue Barriers.

[46] Sample R.A., Nogueira M.F., Mitra R.D., Puram S.V. (2023). Epigenetic regulation of hybrid epithelial-mesenchymal cell states in cancer. Oncogene.

[47] Dobric A., Germain S., Silvy F., Bonier R., Audebert S., Camoin L., Dusetti N., Soubeyran P., Iovanna J., Rigot V. (2025). E-Cadherin Is a Structuring Component of Invadopodia in Pancreatic Cancer. J. Cell Mol. Med..

[48] Haerinck J., Goossens S., Berx G. (2023). The epithelial-mesenchymal plasticity landscape: Principles of design and mechanisms of regulation. Nat. Rev. Genet..

[49] Mayor R., Etienne-Manneville S. (2016). The front and rear of collective cell migration. Nat. Rev. Mol. Cell Biol..

[50] Franklin J.M., Wu Z., Guan K.L. (2023). Insights into recent findings and clinical application of YAP and TAZ in cancer. Nat. Rev. Cancer.

[51] Shi H., Zou Y., Zhong W., Li Z., Wang X., Yin Y., Li D., Liu Y., Li M. (2023). Complex roles of Hippo-YAP/TAZ signaling in hepatocellular carcinoma. J. Cancer Res. Clin. Oncol..

[52] Fu M., Hu Y., Lan T., Guan K.L., Luo T., Luo M. (2022). The Hippo signalling pathway and its implications in human health and diseases. Signal Transduct. Target. Ther..

[53] Tian Z., Xu C., He W., Lin Z., Zhang W., Tao K., Ding R., Zhang X., Dou K. (2023). The deubiquitinating enzyme USP19 facilitates hepatocellular carcinoma progression through stabilizing YAP. Cancer Lett..

[54] Flinn M.A., Otten C., Brandt Z.J., Bostrom J.R., Kenarsary A., Wan T.C., Auchampach J.A., Abdelilah-Seyfried S., O’Meara C.C., Link B.A. (2020). Llgl1 regulates zebrafish cardiac development by mediating Yap stability in cardiomyocytes. Development.

[55] Itoh S., Maeda T., Shimada M., Aishima S., Shirabe K., Tanaka S., Maehara Y. (2004). Role of expression of focal adhesion kinase in progression of hepatocellular carcinoma. Clin. Cancer Res..

[56] Chen J.S., Huang X.H., Wang Q., Chen X.L., Fu X.H., Tan H.X., Zhang L.J., Li W., Bi J. (2010). FAK is involved in invasion and metastasis of hepatocellular carcinoma. Clin. Exp. Metastasis.

[57] Nakabayashi H., Taketa K., Miyano K., Yamane T., Sato J. (1982). Growth of human hepatoma cells lines with differentiated functions in chemically defined medium. Cancer Res..

[58] Stemmer M., Thumberger T., Del Sol Keyer M., Wittbrodt J., Mateo J.L. (2015). CCTop: An Intuitive, Flexible and Reliable CRISPR/Cas9 Target Prediction Tool. PLoS ONE.

[59] Concordet J.P., Haeussler M. (2018). CRISPOR: Intuitive guide selection for CRISPR/Cas9 genome editing experiments and screens. Nucleic. Acids Res..

[60] Doench J.G., Fusi N., Sullender M., Hegde M., Vaimberg E.W., Donovan K.F., Smith I., Tothova Z., Wilen C., Orchard R. (2016). Optimized sgRNA design to maximize activity and minimize off-target effects of CRISPR-Cas9. Nat. Biotechnol..

[61] Han S., Cui Y., Helbing D.L. (2020). Inactivation of Horseradish Peroxidase by Acid for Sequential Chemiluminescent Western Blot. Biotechnol. J..

