# Supplementary material for: Loss of LLGL1 Elevates EGFR/RAS/MAPK Signaling and Remodels EMT Markers in Huh-7 Hepatocellular Carcinoma Cells
Source: Int J Mol Sci. 2026 Mar 24;27(7):2959. doi: 10.3390/ijms27072959 (PMC13073841; doi:10.3390/ijms27072959)
Supplement: Supplementary file 1 [file ijms-27-02959-s001.zip › ijms-4109886-supplementary revised/Fig. S1.pdf]

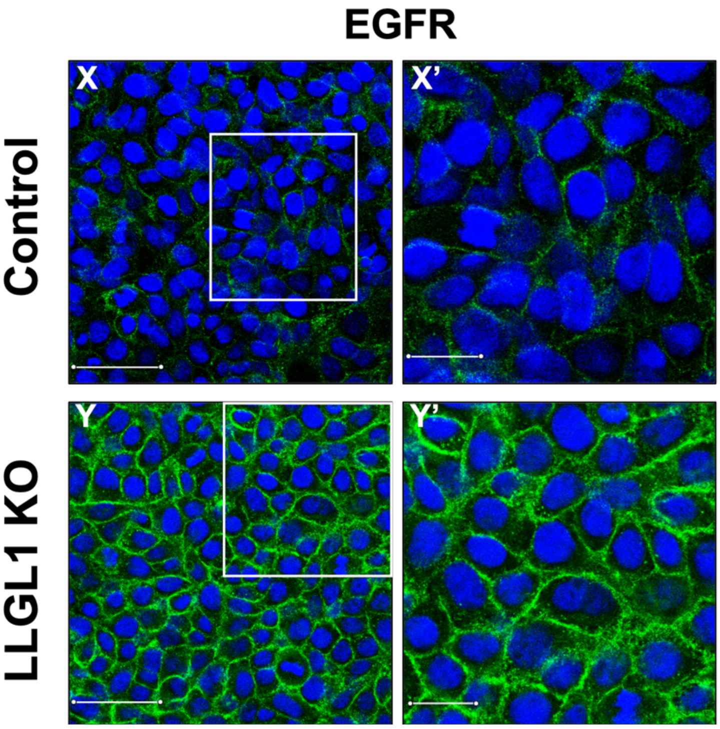

Figure S1. Non-permeabilized IF analysis of EGFR localization in control and LLGL1 KO Huh-7 cells. Representative confocal IF images showing EGFR staining under non-permeabilized conditions in control (X, X′) and LLGL1 KO (Y, Y′) Huh-7 cells. Cells were fixed but not permeabilized prior to antibody incubation to preferentially visualize surface-associated EGFR. White boxed regions in panels X and Y indicate areas shown at higher magnification in panels X′ and Y′, respectively. Images were acquired using a 63× objective under identical imaging settings for both groups. Scale bars: 20 μm.
