# Supplementary material for: Loss of LLGL1 Elevates EGFR/RAS/MAPK Signaling and Remodels EMT Markers in Huh-7 Hepatocellular Carcinoma Cells
Source: Int J Mol Sci. 2026 Mar 24;27(7):2959. doi: 10.3390/ijms27072959 (PMC13073841; doi:10.3390/ijms27072959)
Supplement: Supplementary file 1 [file ijms-27-02959-s001.zip › ijms-4109886-supplementary revised/Table S1 revised.docx]

**Table S1.** List of primary and secondary antibodies.

| **Antibody, clone** | **Company** | | **Catalog No** | **Dilution** | **Application** |
| --- | --- | --- | --- | --- | --- |
| **Primary Antibody** |  |  |  |  |  |
| Anti-Beta Actin, | Abcam | | Ab8226 | 1:5,000 | WB |
| Anti-Claudin-1, D3H7C | CST | | 13995 | 1:1,000 | WB |
| Anti-E-Cadherin, 24E10 | CST | | 3195 | 1:1,000/1:150 | WB/IF |
| Anti-EGFR, A-10 | SCBT | | sc-373746 | 1:1,000/1:150 | WB/IF |
| Anti-ERK1/2, C-9 | SCBT | | sc-514302 | 1:1,000 | WB |
| Anti-LLGL1, D2B5A | CST | | 12159 | 1:1,000 | WB |
| Anti-MEK1/2, L38C12 | CST | | 4694 | 1:1,000 | WB |
| Anti-N-Cadherin, D4R1H | CST | | 13116 | 1:1,000 | WB |
| Anti-pan-RAS | CST | | 8832 | 1:2,500 | WB |
| Anti-phospho-EGFR (Tyr1068) | TFS | | 44-788G | 1:1,000 | WB |
| Anti-phospho-EGFR (Tyr1173) | TFS | | 44-794G | 1:1,000 | WB |
| Anti-phospho-ERK1/2 (Thr202/Tyr204) | CST | | 9101 | 1:2,500 | WB |
| Anti-phospho-MEK1/2 (Ser217/Ser221), 41G9 | CST | | 9154 | 1:1,000 | WB |
| Anti-phospho-RAF1 (Ser338), 56A6 | CST | | 9427 | 1:2,000 | WB |
| Anti-phospho-RSK1-3 (Ser380), D3H11 | CST | | 11989 | 1:1,000 | WB |
| Anti-RAF1, 8H11L22 | TFS | | 702166 | 1:2,000 | WB |
| Anti-RSK1-3, F.940.7 | TFS | | MA5-15040 | 1:2,000 | WB |
| Anti-Snail, C15D3 | CST | | 3879 | 1:1,000 | WB |
| Anti-Vimentin, D21H3 | CST | | 5741 | 1:1,000/1:150 | WB/IF |
| Anti-ZEB1, E2G6Y | CST | | 70512 | 1:1,000/1:150 | WB/IF |
| Anti-ZO-1, D6L1E | CST | | 13663 | 1:1,000 | WB |
| **Secondary Antibody** |  | |  |  |  |
| Anti-mouse IgG-HRP | Bio-Rad | | 1705047 | 1:7000 | WB |
| Anti-rabbit IgG-HRP | Bio-Rad | | 1705046 | 1:7000 | WB |
| Anti-rabbit IgG-Alexa Fluor 647 | Abcam | | ab150083 | 1:750 | IF |
| Anti-mouse IgG-Alexa Fluor 488 | Abcam | | ab150117 | 1:750 | IF |

CST: Cell Signaling Technology, IF: Immunofluorescence, SCBT: Santa Cruz Biotechnology, TFS: Thermo Fisher Scientific, WB: Western blotting.
